# Alternative Zoning Scenarios for Regional Sustainable Land Use Controls in China: A Knowledge-Based Multiobjective Optimisation Model

**DOI:** 10.3390/ijerph110908839

**Published:** 2014-08-28

**Authors:** Yin Xia, Dianfeng Liu, Yaolin Liu, Jianhua He, Xiaofeng Hong

**Affiliations:** 1 School of Resource and Environmental Science, Wuhan University, 129 Luoyu Road, Wuhan 430079, China; E-Mails: linusrain@hotmail.com (Y.X.); yaolin610@126.com (Y.L.); hjhwhu@gmail.com (J.H.); 2 Xiangyang Municipal Bureau of Land and Resources, 13 Xiangcheng Street, Xiangyang 441100, China; 3 Changjiang River Scientific Research Institute, Changjiang Water Resources Commission, 23 Huangpu Road, Wuhan 430010, China; E-Mail: hongxf1215@gmail.com

**Keywords:** land use zoning, scenario, multiobjective optimisation, goal programming, simulated annealing

## Abstract

Alternative land use zoning scenarios provide guidance for sustainable land use controls. This study focused on an ecologically vulnerable catchment on the Loess Plateau in China, proposed a novel land use zoning model, and generated alternative zoning solutions to satisfy the various requirements of land use stakeholders and managers. This model combined multiple zoning objectives,* i.e.*, maximum zoning suitability, maximum planning compatibility and maximum spatial compactness, with land use constraints by using goal programming technique, and employed a modified simulated annealing algorithm to search for the optimal zoning solutions. The land use zoning knowledge was incorporated into the initialisation operator and neighbourhood selection strategy of the simulated annealing algorithm to improve its efficiency. The case study indicates that the model is both effective and robust. Five optimal zoning scenarios of the study area were helpful for satisfying the requirements of land use controls in loess hilly regions, e.g., land use intensification, agricultural protection and environmental conservation.

## 1. Introduction

China has experienced a rapid stage of economic development since the 1990s. The population increase and economic growth have accelerated the need for various land uses [1,2], and intensified the conflicts between urban expansion, cultivated land conservation and agro-ecological environment protection [3]. Thus, it is necessary for the Chinese Government to implement a sustainable policy to regulate landscape and land use patterns [4,5].

Land use zoning is one of the most effective measures to control the various land use activities. It originated for the re-construction of disordered and undisciplined cities like Berlin and later attracted considerable attention around the world [6,7]. Some countries, e.g., Germany, United States and France, have employed municipal/county zoning ordinances to optimise residential, industrial, commercial and ecological land use in rural and urban planning [8,9,10,11], whereas in China, major efforts have been made on regional land use zoning to reconcile the land use conflicts between rural and urban development and protect agricultural land from the occupation of urban expansion [12].

**Figure 1:**
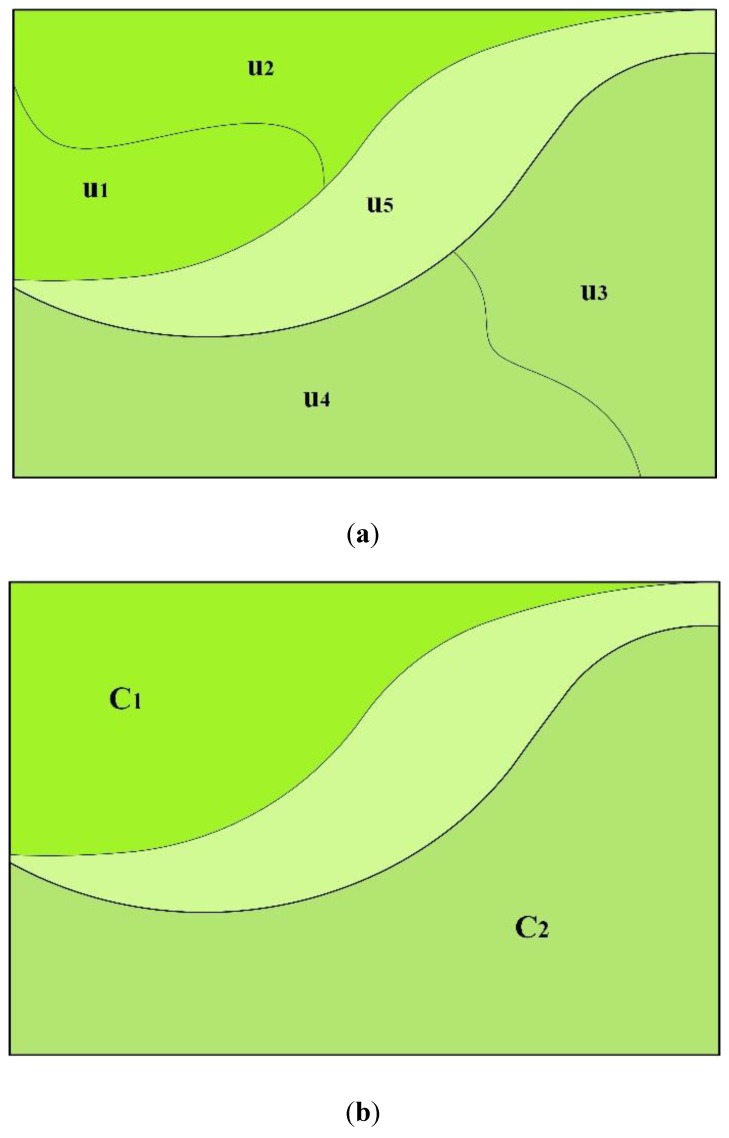
The relationships between a zone, a subregion and a zoning unit. (**a**) The units in a zone; (**b**) The subregions in a zone.

As a geographically contiguous part of administrative division, land use zones are divided based on land quality and natural, social and economic land use conditions, and have their own ordinances that prescribe what types of land use is allowable within them [12,13,14]. These zones bridge the gap between micro and macro land use controls, provide guidance in the case of conflicts between the various land use activities and determine the best land use options in practice [15]. Land use zoning towards sustainable development involves a set of sustainability objectives related to agricultural land prevention, ecological environment restoration, urban sprawl restriction and scattered rural settlement reclamation. Accordingly, nine different types of land use zones have been employed to regulate land use activities at the county scale in China, which is the major scale of Chinese land use planning and management [5,16]. According to the Chinese land use planning outline (2006–2020) at the county scale, these zones contain basic farmland preservation areas (BFPA), general agricultural land (GAL), forestry land (FL), pasture land (PL), urban construction land (UCL), rural construction land (RCL), independent industrial and mining land (IIML), tourism land (TL), and natural and humanistic preservation areas (NHLPA) [17]. Each type of zone is a combination of land units with approximate attribute values and can provide one type of land use regulations to policy makers and land managers. These zones have several characteristics in common, e.g., a zone may comprise some subregions (e.g., $C_{1}$ and $C_{2}$ , separated by the unit $u_{5}$ ) that are not spatially contiguous, but the land units $u_{i}$ within each subregion are compact, and the minimum areas of subregions within each zone are correlated with the spatial scale in a zoning map (Figure 1).

Appropriate zoning techniques can facilitate the determination of land use zones and improve the efficiency of land use management. Current zoning methods are classified into four categories, including spatial overlay analysis, multiple criteria analysis, integer programming and heuristic methods. Early efforts were made on spatial overlay analysis technique, which can aggregate physical and socioeconomic data from other maps to land units and then group the units with homogenous attribute values into different land use zones, but hardly maintain the spatial contiguity and compactness of land use zones [18]. Then, land use patterns can be zoned by an evaluation with a formal statement of the multiple land use priorities as observed from the different viewpoints of all involved stakeholders [19,20]. This type of methods consists of strategic environmental assessment (SEA), statistical analysis (e.g., principle component analysis, discriminant analysis and variance analysis) and spatial clustering methods [21,22]. In terms of spatial clustering, a set of land units can be grouped into various land use zones by comparing multiple land use criteria, and land units within a zone have highly similar land use conditions but are different from the land units of other zones [23]. The aforementioned methods are deterministic and efficient, however, can only produce one zoning solution if given a particular input and hardly handle the complexity and uncertainty of land use systems. 

Integer programming model was first employed by Hess to solve the zoning issue, and then extensive studies improved his model to obtain better zoning solutions [24,25,26]. However, these models cannot afford a heavy computation burden imposed by the combination of multiple zoning objectives and uncertain land use factors. Accordingly, heuristic algorithms were used to solve the complex land use zoning problem [27,28]. Besides the traditional ones, some hybrid heuristic algorithms, performing better than any of their component heuristic algorithms individually, have been constructed to obtain better land use zoning solutions. For example, Liu* et al.* applied an improved multiobjective particle swarm optimisation algorithm equipped with a crossover and a mutation operator to optimise land use zones at the county level in China [29]. Other beneficial attempts in combination with heuristic algorithms include Geographic Information System (GIS) based information flow techniques, visualisation techniques, complex geographical computation models based on cellular automata (CA) techniques and automated land subdivision tools [30,31,32]. In addition, some heuristic methods for spatial land use allocation as well as other zoning issues, e.g., political districting, school redistricting and legislative zoning, are favourable for land use zoning, although they have different zoning variables [33,34,35,36]. The wide applications of heuristic algorithms make it possible to obtain optimal zoning alternatives in a reasonable time and to introduce land use knowledge to improve the rationality of land use zones [37].

The purpose of this research is to propose an optimal land use zoning model and to obtain zoning alternatives at a loess hilly county in China for the sustainable land use decision making. Regarding land use zoning as a nonlinear and multiobjective optimisation problem, we proposed a knowledge-based multiobjective land use zoning model based on goal programming (GP) and a modified simulated annealing (SA) algorithm. The model combined zoning suitability, compatibility with existing land use planning solutions, spatial compactness of land use zones and zoning constraints to describe the zoning problem, and searched for the optimal solutions by using an improved SA algorithm. GP was employed to balance the conflicts between zoning objectives and to produce optimal land use options for land planners under the controls of given goals [38,39]. Meanwhile, land use knowledge was introduced into the solution initialising operator and neighbourhood selecting strategies of the SA algorithm to improve the optimisation efficiency. The remainder of this paper is organised as follows: Section 2 provides a brief introduction of the study area and data. Section 3 proposes a novel zoning model. Section 4 analyses the data and discusses the results, and the final section presents the conclusions.

## 2. Data

### 2.1. Study Area

**Figure 2:**
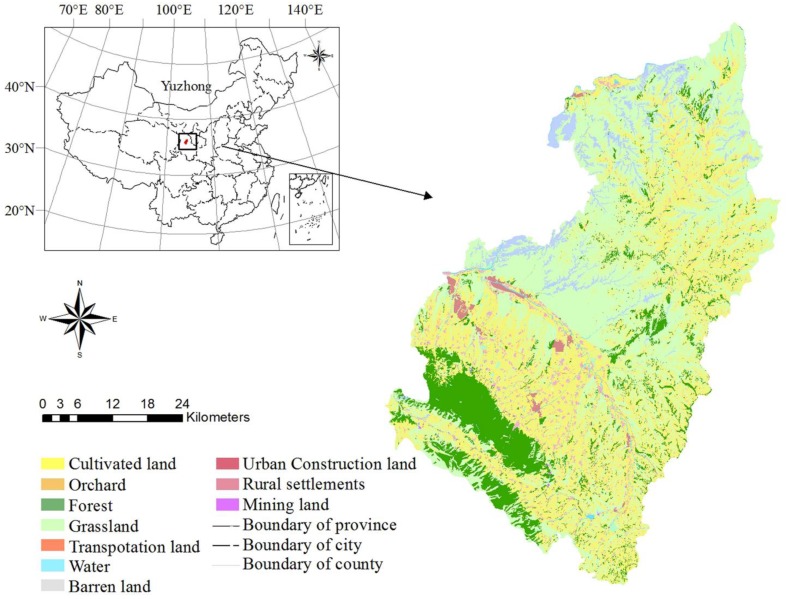
Location of Yuzhong County.

The study area is Yuzhong County in Gansu Province, China. It lies between longitude 103°49′15″E and 104°34′40″E and latitude 35°34′20″N and 36°26′30″N as shown in Figure 2. This area encompasses approximately 329,467.14 hm^2^, has a population of 0.424 million and experiences a typical continental monsoon climate with an average annual precipitation between 250 and 350 mm. Cultivated land, grassland and forest are the three major land use types, comprising 42%, 46% and 11% of the whole area, respectively. The land demands of settlement and subsistence agriculture are increasing rapidly due to the economic development and the population growth. Widespread human-dominated land uses, extensive agriculture and highly erodible loess hill and valley have made this region a conservation focus. Scattered rural settlements in this region also need to be rearranged due to their inconvenient transportation network and poor infrastructure.

### 2.2. Data and Processing

A shapefiles data collection in 2008 at the scale of 1:50,000 was derived from the Lanzhou Municipal Bureau of Land and Resources and the Lanzhou Municipal Bureau of Land Use Planning, including actual land use maps, suitability evaluation maps of each land use zone type, slope maps, natural conservation maps, urban and rural construction land use planning maps and ecological planning maps. 17,578 vector units in the actual land use map at the scale of 1:50,000 were considered as the basic zoning units. The zoning suitability of all units was classified into ten levels from 1 to 10, where 10 denotes the highest suitability level of the units for a certain zone type. The suitability and slope data were assigned to the zoning units through spatial overlay analysis in Arcmap 10 software. We recorded topological relationships between the units by using a timely updated adjacency list, and two units would be regarded as noncontiguous if they shared no more than one vertex. 

According to the guidelines for general land use planning at the county scale in China [17], the study area can be divided into seven land use zones. Table 1 lists the zoning types, area thresholds and the minimum parcel areas of each zone in land use zoning maps at the scale of 1:50,000. The area thresholds of each zone were determined according to the 2006–2020 land use potential assessment data of Yuzhong County, which were acquired from the Yuzhong County Bureau of Land and Resources.

**Table 1:** Descriptions of land use zones in Yuzhong County.

Zone Type	Area Threshold/hm^2^	Minimum Parcel Area/hm^2^
Basic farmland preservation areas (BFPA)	(97,000, 100,000)	5
General agricultural land (GAL)	(23,500, 28,500)	5
Forestry land (FL)	(10,200, 14,800)	25
Pasture land (PL)	(132,500, 137,500)	25
Urban construction land (UCL)	(3200, 3700)	5
Rural construction land (RCL)	(7450, 7850)	5
Natural and humanistic preservation areas (NHLPA)	(29,000, 30,000)	25

## 3. Methodologies

**Figure 3:**
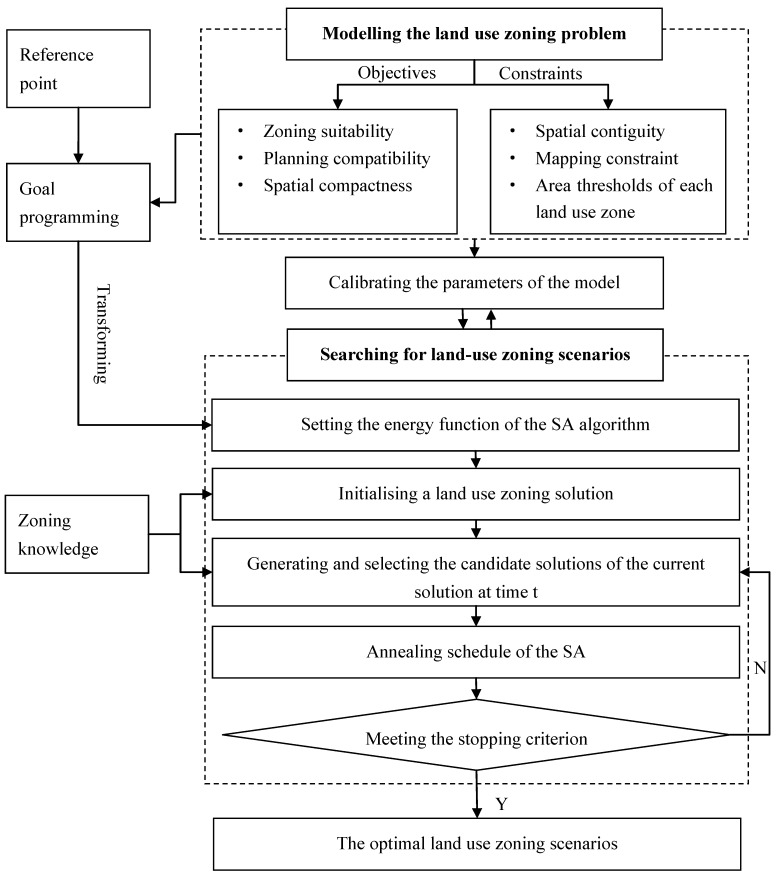
A flow chart of zoning process.

The proposed zoning model comprises two procedures, modelling the land use zoning problems with goal programming techniques, and searching for the optimal zoning solutions by using a modified SA algorithm. Figure 3 displays a flow chart of the zoning process.

### 3.1. Modelling the Land Use Zoning Problem

#### 3.1.1. Zoning Objectives

Sustainable land uses at the loess hilly areas focus on agricultural protection, land use intensification and environmental conservation. Thus, maximum zoning suitability, maximum planning compatibility and maximum spatial compactness serve as three land use zoning objectives.

(1) Maximum zoning suitability

Suitability analysis is a prerequisite of land use planning. The suitability assessment determines the appropriateness of a given unit for a particular zone type and guides the land use based on the evaluated potential of the unit. Assume that $u_{i}$ is a zoning unit, $a_{i}$ is the area of $u_{i}$ , $x_{ik}$ is the suitability value of $u_{i}$ for zone k , $u_{ik}$ is a binary variable, and $u_{ik}=1$ when $u_{i}$ is located within the zone k , or else $u_{ik}=0$ . Let s denote a zoning solution, the suitability of the whole zoning solution is expressed as:

(1) $$f_{1}(s)=\sum_{i=1}^{N}\sum_{k=1}^{K}u_{ik}x_{ik}a_{i},1\leq x_{ik}\leq 10$$

(2) Maximum planning compatibility

Agricultural protection and environmental conservation at the loess hilly areas aim at protecting the arable land and natural resources from the disturbance of rural and urban land use. The existing planning solutions, e.g., basic farmland preservation planning, natural preservation planning and urban planning, can provide guidance for the conservation. Thus, land use zones should keep consistent with these planning solutions. Assume $A_{l}$ denotes the area of the l th existing planning solutions, $O_{l}$ represents the overlapping area between the optimal zoning solution s and the existing planning solution l , the planning compatibility objective minimises the conflicting area:

(2) $$f_{2}(s)=\sum_{l=1}^{L}O_{l}/A_{l}$$

where L is the number of the existing planning solutions. The value of $f_{2}(s)$ ranges from 0 to L , and the smaller the value is, the higher the planning compatibility is.

(3) Maximum spatial compactness

Intensification, especially intensive rural settlement, is another of the essential aspects of sustainable land use in Yuzhong [40]. The decrease of land use fragments can facilitate spatial land use control. Thus, spatial contiguity ( $g_{1k}(s_{k})$ ) and shape index ( $g_{2k}(s_{k})$ ), were employed to measure the spatial compactness of the zone $s_{k}\subset s$ [41].

- $g_{1k}(s_{k})$ represents the spatial contiguity of the zone $s_{k}$ , which can be represented as the number of subregions within the k th zone. The smaller its value is, the more contiguous the k th zone is:
  (3) $$g_{1k}(s_{k})=M_{k}$$
- $g_{2k}(s_{k})$ denotes the shape index of the zone $s_{k}$ , which is the weighted average of the shape index of all subregions within the zone. Let $Area(C_{kj})$ denote the area of the subregion $C_{kj}$ in the zone $s_{k}$ , and $Peri(C_{kj})$ denote the perimeter of the subregion, then the shape index satisfies:
  (4) $$g_{2k}(s_{k})=\sqrt{Area(C_{kj})}/SqrtArea(C_{k})\sum_{j=1}^{M_{k}}Comp(C_{kj})$$
  where $SqrtArea(C_{k})=\sum_{j=1}^{M_{k}}\sqrt{Area(C_{kj})}$ , $Comp(C_{kj})=Peri(C_{kj})/\sqrt{Area(C_{kj})}$ , the value of $Comp(C_{kj})$ equals 4 if the subregion $C_{kj}$ is a square, which is the minimum value, $M_{k}$ denotes the number of subregions within the zone $s_{k}$ .

#### 3.1.2. Constraints

(1) The area thresholds of each land use zone

Land use systems feature randomness and uncertainty. Thus, the controls of various zones on land use should be elastic. Assume $R_{k}$ denotes the total area of the zone k , then $R_{k}$ meets:

(5) $$\alpha_{k}\leq R_{k}\leq \beta_{k}\begin{array}{cc} & \forall k=1,2...,K \end{array}$$

where $\alpha_{k}$ and $\beta_{k}$ are the bottom and the upper boundaries of $R_{k}$ as shown in Table 1, respectively.

(2) The minimum parcel areas in land use zones

The land use planning maps at the county scale regulate the minimum parcel areas of different land use zones (Table 1). The parcel whose area is less than the threshold values should be merged into its adjacent parcel with the same zone type. Let $\theta_{k}$ denote the minimum parcel area in the zone k , then the constraint can be represented as:

(6) $$Area(C_{kj})\geq \theta_{k}\begin{array}{cc} & \forall k=1,2...,K,\forall j=1,2...,M_{k} \end{array}$$

(3) Spatial constraints

- Water areas will not be grouped into any zones.
- Zoning types of the built-up areas located at the central counties and towns will be predetermined as UCL or RCL.

Land use compatibility in a zone can provide land use priority and limitation information so as to guide sustainable land use activities [12,13,14,29]. Accordingly, we defined the dominant, allowable and prohibited land use types for each land use zone based on the actual conditions of Yuzhong County (Table 2).

**Table 2:** The dominant, allowable, and prohibited land use types for each land use zone.

Land Use Type	BFPA	GAL	FL	PL	UCL	RCL	NHLPA
Cropland	+	+	±	−	−	−	−
Garden	±	+	±	−	−	−	±
Pasture	−	±	±	+	−	−	±
Forest	−	±	+	±	±	±	±
Transportation	±	±	±	±	+	+	±
Urban settlements	−	−	−	−	+	−	−
Rural settlements	−	±	±	±	−	+	±
Tourism land	−	−	−	−	−	−	+
Barren land	−	−	±	−	±	±	±

Notes: +, dominant land use; ±, allowable land use; −, prohibited land use.

#### 3.1.3. The Land Use Zoning Model

The goal programming method equipped with the reference point technique is employed to describe the multiobjective function F(s) of the zoning problem [42]. Attribute constraints, *i.e.*, Equations (5) and (6), are incorporated into the function F(s) as the “penalty function” [43], and spatial constraints are integrated into the SA algorithm. Then, the land use zoning model can be represented as:

(7) $$\begin{array}{cc} MINIMISE & F(s)=\sum_{p=1}^{P}{\left(\frac{f_{p}(s)-I_{p}}{U_{p}-I_{p}}\right)}^{e}+\sum_{q=1}^{Q}\sum_{k=1}^{K}{\left(\frac{g_{qk}-I_{qk}}{U_{qk}-I_{qk}}\right)}^{e}\;+Penalty(s) \end{array}$$

where $I_{p}$ and $I_{qk}$ are the ideal fitness values of $f_{p}(s)$ and $g_{qk}(s)$ , respectively. $U_{p}$ and $U_{qk}$ are the expected fitness values of $f_{p}(s)$ and $g_{qk}(s)$ fixed by decision makers, respectively, and it holds that $U_{p}>I_{p}$ and $U_{qk}>I_{qk}$ . P and Q represent the number of optimisation objectives. K is the number of zoning types in the zoning solution s . The constant parameter e is the marginal penalty coefficient of the objectives for the deviation of their fitness values from the expected values. The higher the deviation is, the larger the penalty coefficient. 

The penalty function of the zoning solution s can be calculated as:

(8) $$Penalty(s)=\sum_{k=1}^{K}\left(Penalty(R_{k})+Penalty(C_{k})\right)$$

where $Penalty(R_{k})$ and $Penalty(C_{k})$ denote the penalty functions of the quantitative land use structure and the mapping area constraints of the k th zone, respectively:

(9) $$Penalty(R_{k})=(\frac{Max(0,R_{k}-\beta_{k})}{\theta_{k}})+(\frac{Max(0,\alpha_{k}-R_{k})}{\theta_{k}})$$

(10) $$Penalty(C_{k})=\sum_{j=1}^{M_{k}}\frac{Max(0,\theta_{k}-Min(Area(C_{kj}))}{\theta_{k}}$$

After running the model 100 times, the ideal values of $f_{1}(s)$ and $f_{2}(s)$ equal to 2,684,956.6 and 0.0, and the worst values equal to 2,000,000.0 and 1.0, respectively. The extreme values of $g_{1k}(s)$ and $g_{2k}(s)$ for each type of zones are list in Table 3. These values favour the decision makers adjusting expected values, $U_{p}$ and $U_{qk}$ , of each objective to obtain land use zoning alternatives.

**Table 3:** The extreme fitness values of spatial compactness objectives.

Objective	Ideal Values	Worst Values						
		BFPA	GAL	FL	PL	UCL	RCL	NHLPA
*g_1k_*(*s*)	1.0	1000.0	800.0	200.0	200.0	50.0	600.0	10.0
*g_2k_*(*s*)	4.0	20.0	20.0	30.0	70.0	15.0	15.0	30.0

### 3.2. Searching for Land Use Zoning Scenarios

Simulated annealing (SA) is a generic probabilistic metaheuristic for global optimisation problems and is capable of escaping local optima [44]. A modified spatial SA algorithm was employed to search for the optimal land use zoning solutions. The algorithm started from an original zoning solution and improved the solution iteratively until a stopping condition was met or a solution with the minimum/maximum energy was found. In the following, we describe three major procedures of the SA algorithm, including the initialisation of zoning solutions, generation and selection of candidate solutions and annealing schedule.

#### 3.2.1. Initialising a Zoning Solution

Assume $P_{k}(u_{i})$ denotes the probability of the zoning unit $u_{i}$ being grouped into the zone k , then its value is determined by the zoning suitability of $u_{i}$ , current land use type of $u_{i}$ , the existing planning solutions and spatial compactness simultaneously:

(11) $$P_{k}(u_{i})=p_{suit}^{k}(u_{i})\cdot p_{coor}^{k}(u_{i})\cdot p_{comp}^{k}(u_{i})\cdot p_{const}^{k}(u_{i})$$

where: 

$p_{suit}^{k}(u_{i})=\frac{x_{ik}-MIN_{suit}}{MAX_{suit}-MIN_{suit}}$ , denotes the normalised suitability value of $u_{i}$ to the zone k , the constants $MIN_{suit}$ and $MAX_{suit}$ denote the minimum and maximum of the suitability level, equaling to 1 and 10, respectively;

$p_{comp}^{k}(u_{i})=\frac{(g_{2k}(s_{k}+u_{i})-g_{2k}(s_{k}))-MIN^{\bar{K}}g_{2k}}{MAX^{\bar{K}}g_{2k}-MIN^{\bar{K}}g_{2k}}$ , represents the ability of $u_{i}$ to improve the spatial compactness of the zone k ; $g_{2k}(s_{k}+u_{i})$ is the compactness of the zone k if the unit $u_{i}$ is grouped into the zone k ; $\bar{K}$ is the amount of the adjacent zones of the unit $u_{i}$ , and if $\bar{K}=0$ , then $p_{comp}^{k}(u_{i})=1$ , which means that all the units close to $u_{i}$ have not been assigned a zone type;

$p_{coor}^{k}(u_{i})$ equals 1 if the zone type of the unit $u_{i}$ is k and consistent with the existing planning solutions, and 0 otherwise;

$p_{const}^{k}(u_{i})$ equals 1 if the unit $u_{i}$ is within the zone k and consistent with the spatial constraints, and 0 otherwise.

Then, an initial zoning solution is generated based on the seeded region growing method instead of a random technique [45]:

Step 1: Select an ungrouped unit $u_{i}$ as a seed point randomly. Calculate the probability $P_{k}(u_{i})$ , and then determine the zone type of the unit $u_{i}$ by using the roulette method.
Step 2: Select an ungrouped unit $u_{j}$ closed to $u_{i}$ . Calculate the probability $P_{k}(u_{j})$ of the unit $u_{j}$ within an arbitrary zone k , and then determine its zone type by using the roulette method.
Step 3: Search the adjacent units of $u_{j}$ , iterating step 2 greedily until no ungrouped units exists or the subregion with the seed point $u_{i}$ is surrounded by units from different types of zones, and then turn to step 1.

#### 3.2.2. Generating and Selecting Candidate Solutions

The candidate solutions sit within the neighbourhood of the current solution s at the i th iteration of the SA algorithm and impact the optimisation efficiency significantly [46]. At each iteration, the candidate solutions were generated by randomly reshuffling zoning patterns of the current solution s . Let $u_{i}$ represent a boundary unit of the subregion $C_{kj}\subset s$ , and the subregion $C_{k\prime j\prime }\subset s$ is close to the unit $u_{i}$ ( k′≠k ). The change of zone type of $u_{i}$ from k to k′ will cause the generation of a new zoning solution s′ , *i.e.*, one of candidate solutions within the neighbourhood of s . Accordingly, there are six transformation types from the current solution s to its neighbour s′ as illustrated in Table 4. 

**Table 4:** The status of $C_{kj}$ and $C_{k\prime j\prime }$ after the transformation of s to its neighbor s′ .

Type	${C_{kj}}^{*}$	${C_{k\prime j\prime }}^{*}$	Generating New Subregion
Type1	SI	SI	No
Type2	DI	SI	No
Type3	SE	SI	Yes
Type4	SI	ME	No
Type5	DI	ME	No
Type6	SE	ME	Yes

Notes: SI, still independent; DI, disappeared; SE, separated into several small subregions; ME, merged with other subregions of the same zoning type.

**Figure 4:**
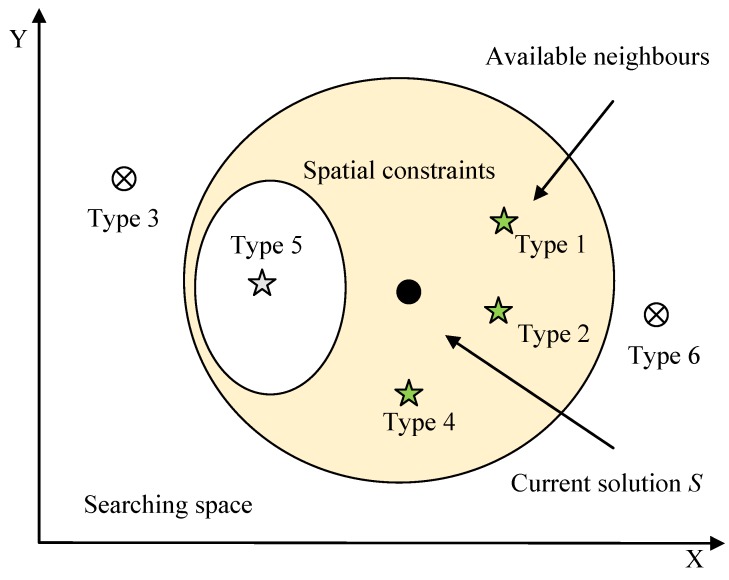
Selection of the acceptable candidate solutions.

The candidates are firstly filtered based on the spatial zoning constraints and the land use knowledge on the compactness and easy implementation of each zone in practice. Type 1, 2, 4 and 5 do not produce any new subregions after the transformation, but type 3 and 6 are the opposite. Together with the consideration of the spatial zoning constraints, only types 1, 2 and 4 are available candidates as shown in Figure 4. Then, the energies of three types of newly generated solutions, * i.e.*, fitness values of objective function F(s) , are compared with that of the current one. The newly generated solutions will be accepted if their fitness values are better than that of the current solution. Otherwise, the algorithm can only accept the new solutions with some probability, depending on the temperature and how much worse it is than the current solution (Metropolis rules). If none of candidates is accepted, the current solution will remain unchanged in the next iteration. 

The accepting probability of candidate solutions satisfies:

(12) $$p=\{\begin{array}{l} \begin{array}{ccc} 1 & if & \Delta E<0 \end{array}\\ \begin{array}{ccc} \exp(-(\Delta E)/T) & if & \Delta E>0 \end{array} \end{array}$$

where ΔE represents the energy deviation between the current solution and the candidate one, T is the temperature at current iteration.

#### 3.2.3. Annealing Schedule

The annealing schedule specifies how the temperature is reduced as the search progresses. Three important parameters, including initial temperature $T_{0}$ , searching time for each temperature L and cooling coefficient ρ∈(0,1) , need to be fixed. It is recommended that the initial temperatureis high enough and decreases as slowly as possible. In this research, we compared four sets of above parameters and employed a geometric cooling technique to decrease the temperature (Table 5). Annealing was halted when fewer than five solutions with worse fitness values had been accepted during the iterations.

**Table 5:** Parameter combinations of the SA algorithm.

Parameter Sets	*T_0_*	*L*	*ρ*
A	1	200	0.8
B	1	1000	0.8
C	1	200	0.2
D	1	1000	0.2

## 4. Results and Discussion

### 4.1. Sensitivity Analysis of the Parameters

**Figure 5:**
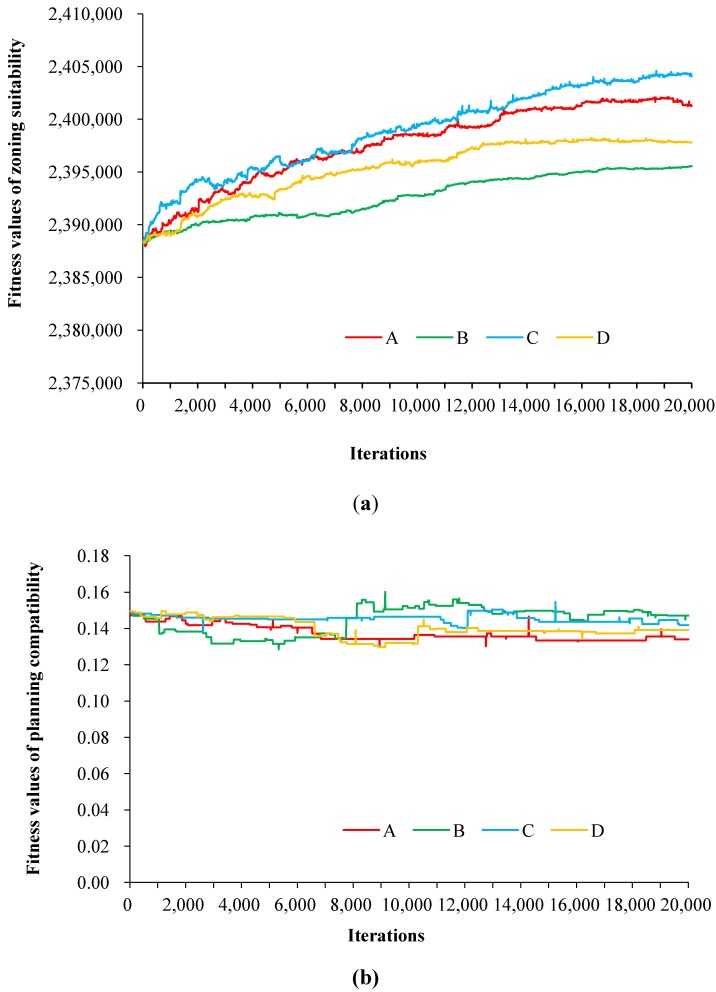
Convergence curves of (**a**) the zoning suitability and (**b**) planning compatibility objective.

Without loss of generality, the expected values of all the objectives were set to 50% of their ideal values, and the total fitness $F(s_{0})$ of the initial solution $s_{0}$ was fixed to 423.41, then the effects of four parameter sets on the zoning solutions were compared. Figure 5 illustrates the convergence processes of the objectives of land suitability and planning compatibility under different parameter scenarios. It can be observed that combination C always obtains higher suitability than A, D and B, and combination B can find out the optimal solution with the highest planning compatibility when the zoning optimisation is terminated. The convergence processes reveal that the parameters are highly correlated with the fitness values of the suitability, whereas uncorrelated with the planning compatibility. During the optimisation, the relationship between the suitability of the zoning solution and L is negative, and if L is fixed, the smaller *ρ* is, the higher zoning suitability would be.

**Figure 6:**
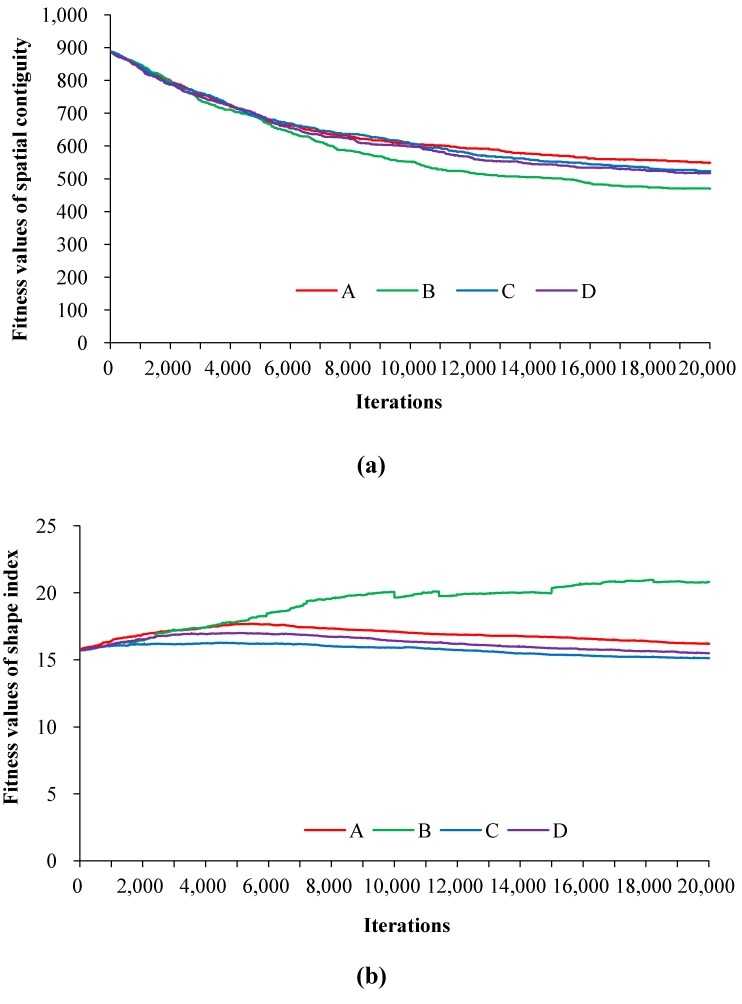
The objective fitness values during the optimisation of basic farmland preservation zone: (**a**) spatial contiguity; (**b**) shape index.

Taking the basic farmland preservation zone as an example, the effects of the parameters on the spatial compactness are depicted in Figure 6. Obviously, combination B outperforms other parameter sets, and obtains less amount and more regular subregions in the basic farmland preservation zone. When the fitness values of the spatial compactness objectives at the 5000th, 10,000th, 15,000th and 20,000th steps are compared, it can be observed that combination B and D generate more spatially contiguous zoning patterns, while curves C and D obtain more regular land use zones. The results imply that the spatial contiguity is positively correlated with *L*, whereas the shape index is negatively correlated with *ρ*. 

**Figure 7:**
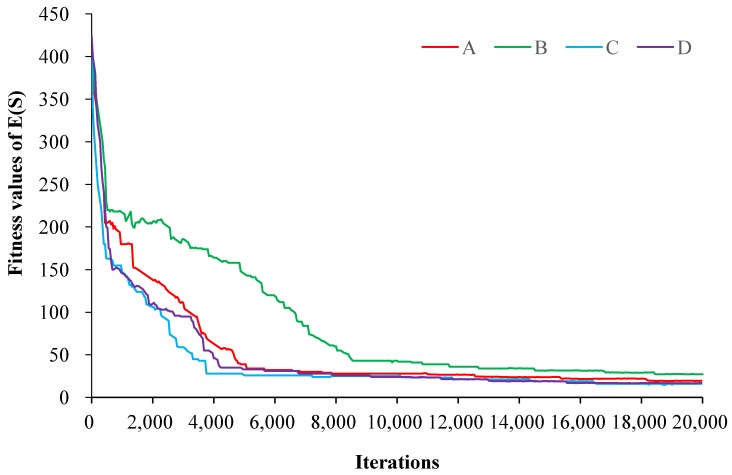
Convergence process of the objective function E(s) under different parameter combinations.

Figure 7 describes the fitness values of the combined objective function E(s) under different parameter combinations. The four curves have different convergence rates at the beginning of the optimisation process and progressively approximate to each other in the end. The optimal fitness values and convergence rates of C and D are very close, and both of them are smaller than those of A and B (Table 6). This implies that cooling parameter ρ has a more significant effect on the search process than parameter *L*. Actually, a small cooling parameter may cause the algorithm to be trapped in local minima, but fortunately, the similar situation did not occur in this research [47]. 

**Table 6:** Optimal fitness values of E(s) and convergence rates of the SA under different parameter combinations.

Parameter Combinations	Optimal Fitness Values	Convergence Rates
A	19.213	14,677
B	27.079	18,299
C	16.331	13,914
D	16.429	13,530

### 4.2. Alternative Land Use Zoning Scenarios

Five zoning scenarios driven by different optimisation objectives were simulated for Yuzhong county. Scenario 1 underlines the land use suitability, scenario 2 highlights the planning compatibility, scenario 3 and 4 emphasizes the spatial contiguity and the shape index of each zone, respectively, and scenario 5 balances all the objectives to simulate the complexity of the land use system. In the experiment, the expected fitness values of the objectives were set unequally as shown in Table 7, and parameter combination C was used to obtain zoning scenarios according to the sensitivity analysis of the parameters.

**Table 7:** Expected fitness values (%) of the optimisation objectives under different zoning scenarios.

Zoning Scenario	*U*_1_	*U*_2_	*U*_11_	*U*_12_	*U*_13_	*U*_14_	*U*_15_	*U*_16_	*U*_17_	*U*_21_	*U*_22_	*U*_23_	*U*_24_	*U*_25_	*U*_26_	*U*_27_
1	80	20	50	50	50	50	50	50	50	50	50	50	50	50	50	50
2	20	80	50	50	50	50	50	50	50	50	50	50	50	50	50	50
3	50	50	80	80	80	80	80	80	80	20	20	20	20	20	20	20
4	50	50	20	20	20	20	20	20	20	80	80	80	80	80	80	80
5	75	90	60	60	60	60	75	75	60	60	60	60	60	75	75	60

Table 8 shows the achieved fitness values of the objectives under different zoning scenarios. The achieved value of the land use suitability in scenario 1 is 81.1%, 1.3%, 4.1%, and 0.4% higher than that in scenarios 2, 3, 4 and 5, respectively, which is consistent with the preference of the suitability objective. The achieved value of the planning compatibility in scenario 2 is 6.6%, 4.4%, 6.7% and 1.8% higher than that in scenario 1, 3, 4 and 5, respectively. In terms of the spatial compactness, the achieved values of the spatial contiguity in scenario 3 increase by 13.0%, 15.3%, 49.4% and 9.6% on average compared with those in scenario 1, 2, 4 and 5, and the achieved values of the shape index in scenario 4 increase by 18.0%, 18.0%, 32.3% and 8.0% on average, compared with those in scenarios 1, 2, 3 and 5, respectively. Scenario 5 emphasizes high expectation for land use suitability, planning compatibility, spatial contiguity and the shape index of basic farmland protection region simultaneously. As a result, the achieved fitness values of the objectives increase remarkably in comparison with those in other scenarios. 

An overlay analysis was performed to compare five zoning solutions. Only slight changes related to the suitability objective are observed in the results, and the reason is that land use suitability has been taken into account in the initialisation of the zoning scenarios. In terms of the planning compatibility, all the scenarios are approximately consistent with the existing planning solutions, and especially in scenario 4, the achieved fitness values reach up to 90% of the ideal values. From the perspective of spatial pattern, the shapes of some subregions in scenario 3, e.g., basic farmland preservation areas, are irregular and banding distributed compared with the subregions in scenario 4, but the number of land use patches decreases by 5.2%. The differences between five scenarios prove that policy makers can integrate participatory preferences into the regional land use planning to regulate the various land use activities by adjusting the expectation of the zoning objectives.

**Table 8:** Achieved fitness values (%) of the objectives under different zoning scenarios.

Zoning Scenario	*f*_1_	*f*_2_	*g*_11_	*g*_12_	*g*_13_	*g*_14_	*g*_15_	*g*_16_	*g*_17_	*g*_21_	*g*_22_	*g*_23_	*g*_24_	*g*_25_	*g*_26_	*g*_27_
1	56.5	85.1	42.6	53.8	55.8	46.2	49.0	33.7	66.7	25.4	54.1	61.0	37.8	47.1	53.4	45.8
2	31.2	90.7	43.3	52.9	53.0	47.1	49.2	30.5	65.4	25.6	54.3	60.8	37.5	47.4	52.6	45.9
3	55.8	86.9	56.9	60.1	56.8	52.3	51.8	35.4	79.9	18.9	51.5	58.8	37.2	40.5	51.3	40.7
4	54.3	85.0	25.7	42.6	44.7	40.7	30.6	27.5	61.5	37.5	62.1	64.9	44.2	52.0	57.1	56.2
5	56.3	89.1	42.7	56.1	55.3	51.8	50.1	34.6	68.2	35.9	61.7	59.8	39.1	49.4	54.9	46.7

### 4.3. Analysis of the Sustainable Zoning Solutions

**Figure 8:**
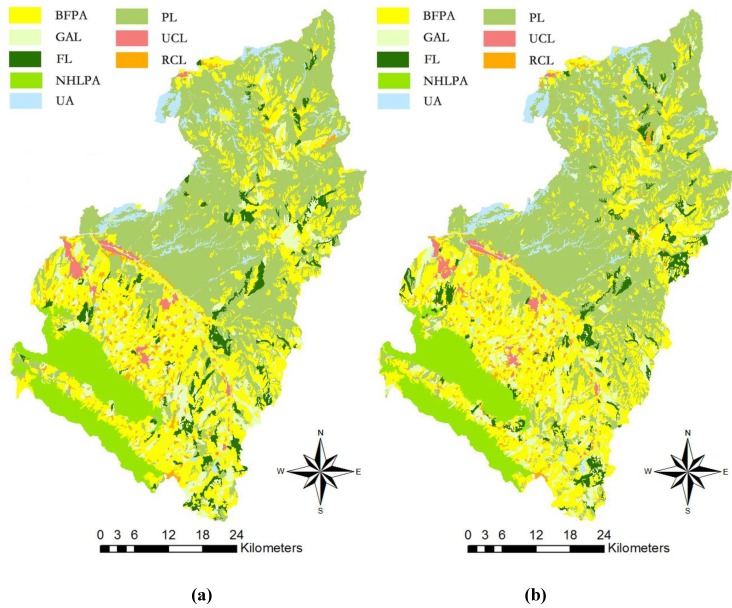
Land use zones for Yuzhong County: (**a**) optimal solution; (**b**) initial solution.

Scenario 5 was selected as a Pareto optimal solution and compared with the initial zoning pattern obtained by using the seeded region growing method (Figure 8). We measured the improvement of the zoning solution from the perspectives of zoning suitability, planning compatibility and spatial compactness in the following.

The results illustrate that the suitability levels for forest, pasture and urban and rural construction areas in the optimal solution increase significantly, except for those of basic farmland preservation zones (Table 9). The initial solution encourages agricultural production and highlights the suitability of basic farm protection areas and general farmland areas, whereas the optimal solution balances the demands of agricultural production, land use intensification and ecological protection of Yuzhong County.

**Table 9:** Suitability values of the initial and optimal solutions for each type of zones.

Zone Types	Suitability Values of the Initial Solution	Suitability Values of the Optimal Solution	Change Ratios (%)
BFPA	622,234.0	615,548.0	−1.07
GAL	126,403.0	118,490.0	−6.26
FL	77,695.0	83,082.0	6.93
PL	1,174,396.0	1,190,378.0	1.36
UCL	27,979.0	28,283.0	1.10
RCL	49,859.0	51,187.0	2.66
NHLPA	903,677.0	903,685.0	0.00

**Figure 9:**
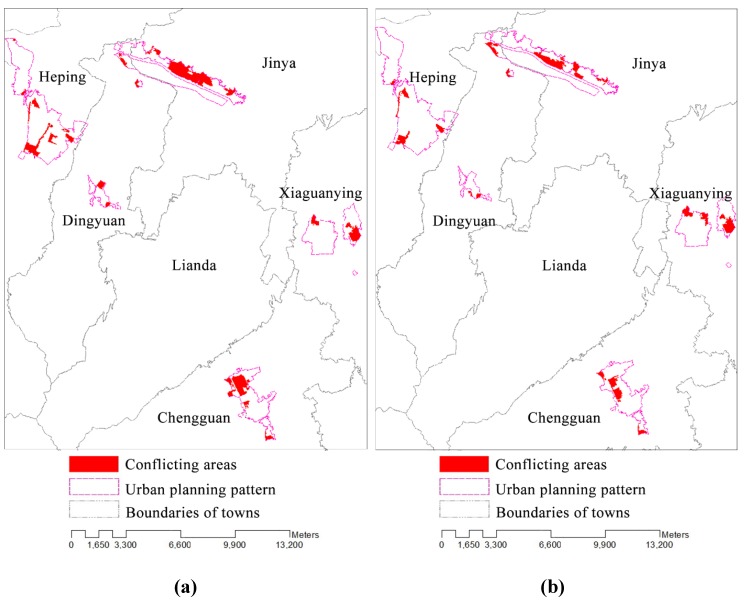
Conflicting regions of the optimal (**a**) and the initial (**b**) land use zones with the existing planning solutions.

**Figure 10:**
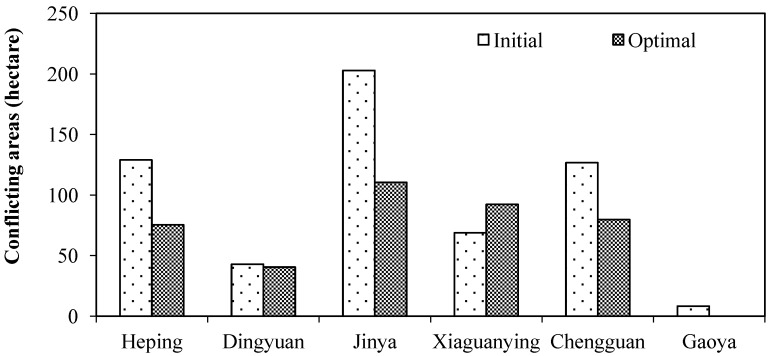
Conflicting areas for all the towns in Yuzhong County.

Figure 9 shows the conflicting areas between the zoning solutions and the existing urban and rural construction land plans the proportion of which reaches up to 15.88% in the initial solution and 12.26% in the optimal solution. The conflicting areas are scattered throughout Heping Town, Jinya Town, Dingyuan Town, Xiaguanying Town and Chengguan Town. The maximum difference emerges in Jianya Town, reaching up to 92.41 hectares, followed by Heping Town and Chengguan Town, reaching up to 53.68 and 46.89 hectares, respectively. To improve the agglomeration effects of the scattered rural settlements, the optimal solution decreases the conflicting areas of all of the towns, except for Xiaguanying Town (Figure 10). 

The spatial compactness of each zone or subregions is depicted by the spatial contiguity and the shape index. The results show that the optimal solution improves the spatial contiguity and shape regulation of most zones remarkably.

**Figure 11:**
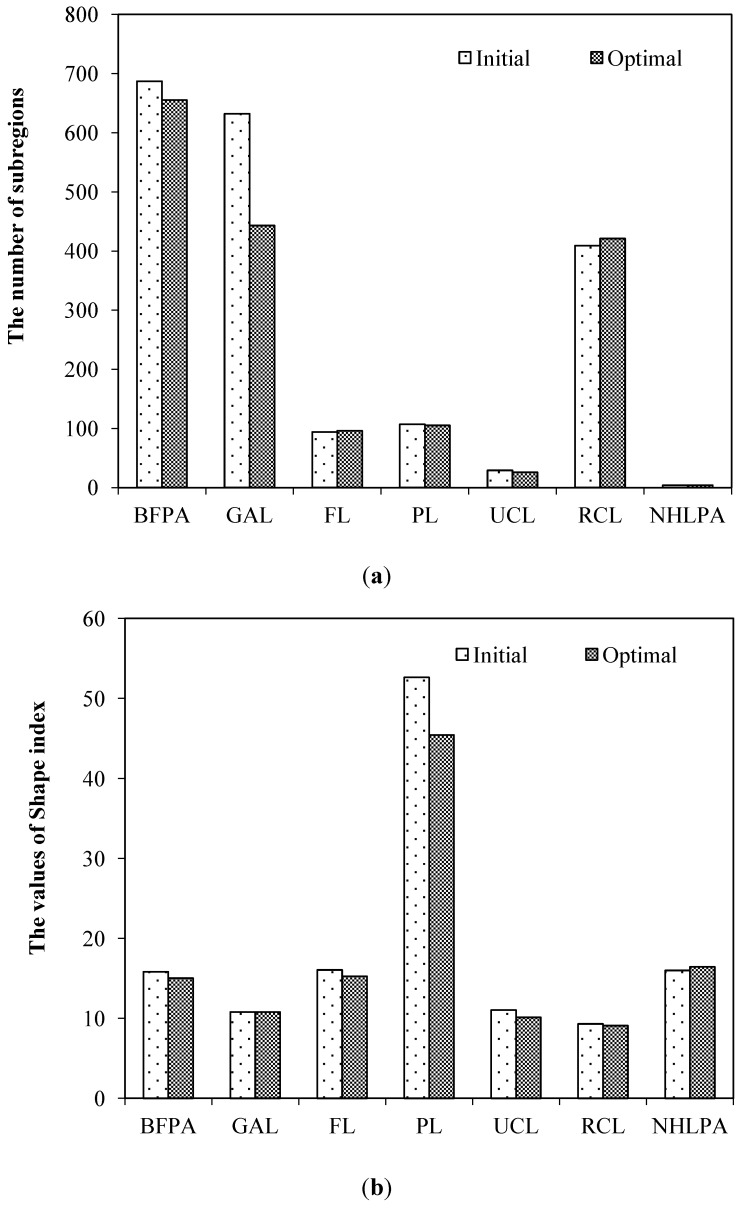
The comparison of spatial compactness between the initial and the optimal zoning solution: (**a**) spatial contiguity; (**b**) shape index.

Figure 11a shows the number of subregions in each zone of the optimal solution and the initial solution. The optimal solution decreases the zoning fragmentation except for forestry areas and urban and rural construction areas. The number of subregions in general farmland zone decreases by 189, and the number in basic farmland protection zone decreases by 32. The changes of the shape index of each zone are shown in Figure 11b. Obviously, lower shape index values emerge in most zones of the optimal solution, except general farmland areas and natural conservation areas. The shape index of the pasture zone displays the greatest decrease, reaching up to 13.60%, while those of urban construction land and basic farmland preservation areas decline by approximate 8.50% and 5.15%, respectively.

**Figure 12:**
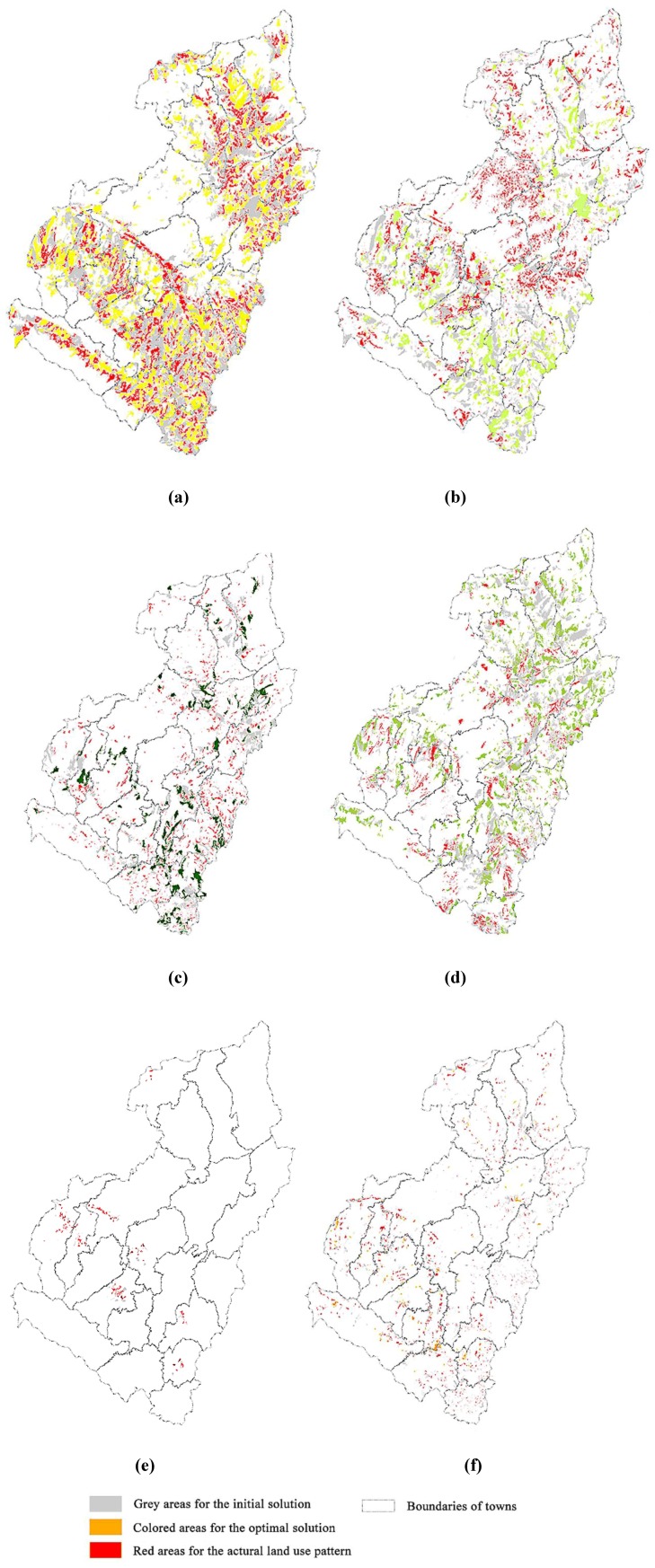
Comparative analysis between the optimal, the initial solutions and the actual land use pattern for each zone: (**a**) BFPA; (**b**) GAL; (**c**) FL; (**d**) PL; (**e**) UCL; (**f**) RCL.

Yuzhong County is now at a crucial stage of economic development, and it is necessary to protect agricultural land and ecosystem and to enhance land use intensification due to its fragile natural environment [48]. Effective information regarding environmental responses to future land use as the optimal land use zones obtained by the proposed model provides useful support for decision making in this context [49]. For instance, basic farmland preservation areas are primarily located in the central and southern parts of Yuzhong County, possessing good agricultural production conditions. General agricultural areas are mainly located in the northeastern, central, and northern part of Yuzhong County, providing supplementary agricultural production in addition to basic farmland. Urban construction areas expand based on the built-up areas within eight towns, e.g., Chengguan, Heping, and Xiaguanying Town, revealing a compact spatial pattern. Rural construction areas are mainly distributed in central and southern Yuzhong County, and a small amount is scattered in the north. Forestry areas are dispersed within the whole region and comprise the ecological corridors with pasture land in the northern part of the county and nature reservation areas in the south [34]. Compared with the actual land use pattern and the initial zoning solution (Figure 12), the areas of non-construction zones increase significantly in the optimal solution, e.g., basic farmland preservation areas, general farmland, forestry and pasture areas, which is consistent with the effects of the “Grain for Green Project” and the agrarian restructuring process in Northwest China [50,51]. The spatial compact urban and rural construction zones in the optimal solution are required for agrarian restructuring, and this may favour the reclamation of hollowed and scattered villages and the improvement of land use intensification [52].

**Figure 13:**
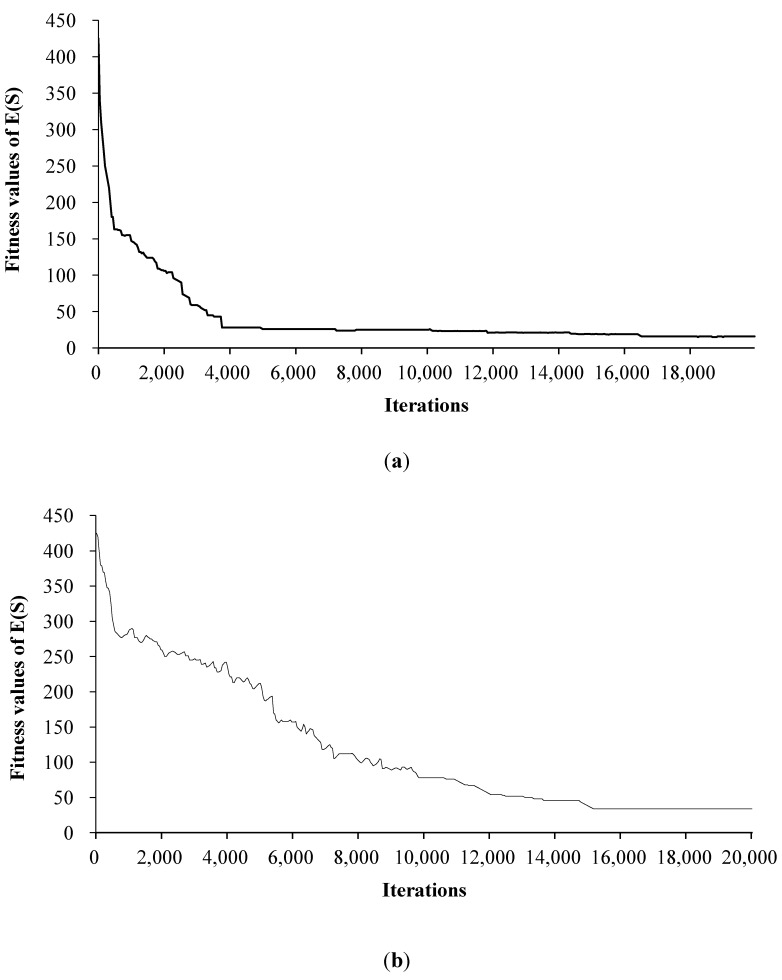
Comparative analysis between (**a**) the modified SA and (**b**) the standard SA.

The results illustrate that the application of the multiobjective land use zoning model works quite well in this study. The main advantage of the model is the generation of land use zoning alternatives, thereby satisfying the various requirements of land planners. This model enables us to deal with land use knowledge to improve the searching efficiency for the optimal zoning solutions. With the application of the objective function F(s) , the comparison of the modified simulated annealing algorithm with the standard one reveals better convergence ability and faster convergence rate of this model (Figure 13). Sensitivity analysis of the parameters and generation of zoning scenarios validate the robustness of the model, proving its availability in the practical applications.

## 5. Conclusions 

This research integrates goal programming and a modified simulated annealing algorithm with a land use zoning problem to obtain alternative zoning solutions for policy makers. Accordingly, a knowledge-based multiobjective land use zoning model was proposed. The model takes land use suitability, planning compatibility and spatial compactness as the zoning objectives and employs goal programming technique to handle the multiple objectives. Some modifications are applied to improving the operators of the SA algorithm, e.g., initialising the zoning solutions by using the seeded region growing method and selecting the candidate solutions based on the land use zoning knowledge.

The experimental results illustrate that the model is applicable and robust. Alternative land use zoning scenarios were generated based on participatory preferences of planners and stakeholders, which can provide guidance for various land use controls. For instance, scenario 5 of Yuzhong county satisfies the requirement of agriculture protection, ecosystem conservation and land use intensification simultaneously. Meanwhile, the sensitivity analysis of model parameters reveals its effects on the optimal zoning solutions, e.g., the negative relationship between the suitability and L , the negative relationship between shape regulation of zones and ρ , and the positive relationship between the spatial contiguity and L *etc.*, and the results aid in the practical application of the model.

Although the proposed model is effective for land use zoning, the model possesses several limitations for applications. First, we only consider some of major zoning objectives, including zoning suitability, planning compatibility and spatial contiguity. However, the conflicts between different land use stakeholders are complex in practice. The interactions among various land use stakeholders can be incorporated into the model to assist planners to determine land use zones. Second, sufficient explorations of the SA algorithm are required to obtain the optimal zoning solutions, but impose a heavy computation burden. Parallel computing technique can be integrated with the model to increase its efficiency. Thus, future works should focus on the integration of the model with land use behaviors and the parallelization of the model.
